# When blood pressure refuses to budge: exploring the complexity of resistant hypertension

**DOI:** 10.3389/fcvm.2023.1211199

**Published:** 2023-06-21

**Authors:** Meeti Keyur Champaneria, Rushi Sanjaykumar Patel, Terry L. Oroszi

**Affiliations:** Department of Pharmacology & Toxicology, Boonshoft School of Medicine, Wright State University, Dayton, OH, United States

**Keywords:** resistant hypertension, medications, lifestyle modification, adherence, mineralocorticoid receptor antagonists, renal denervation, renin- angiotensin-aldosterone system, sleep apnea

## Abstract

Resistant hypertension, defined as blood pressure that remains above goal despite using three or more antihypertensive medications, including a diuretic, affects a significant proportion of the hypertensive population and is associated with increased cardiovascular morbidity and mortality. Despite the availability of a wide range of pharmacological therapies, achieving optimal blood pressure control in patients with resistant hypertension remains a significant challenge. However, recent advances in the field have identified several promising treatment options, including spironolactone, mineralocorticoid receptor antagonists, and renal denervation. In addition, personalized management approaches based on genetic and other biomarkers may offer new opportunities to tailor therapy and improve outcomes. This review aims to provide an overview of the current state of knowledge regarding managing resistant hypertension, including the epidemiology, pathophysiology, and clinical implications of the condition, as well as the latest developments in therapeutic strategies and future prospects.

## 1. Introduction

Resistant hypertension is a chronic condition characterized by persistently high blood pressure that remains above the target levels despite using three or more antihypertensive medications (1, 2). A considerable proportion of the global population is affected by this ailment, which imposes a substantial strain on healthcare systems because of its related complexities and heightened likelihood of morbidity and mortality.

The global burden of resistant hypertension is substantial, with an estimated 100–500 million people affected worldwide (2). The prevalence of resistant hypertension varies depending on several factors, including age, sex, race, ethnicity, and comorbid medical conditions (3). It is more prevalent in older adults, men, and individuals with obesity, diabetes, or kidney disease.

Additionally, certain racial and ethnic groups, such as African Americans and Hispanics, have a higher prevalence of resistant hypertension than others (4). Resistant hypertension is a significant public health concern due to its high prevalence and impact on overall health outcomes. At the same time, accurately determining the prevalence of resistant hypertension is a challenging task, as it requires the exclusion of pseudo-resistance (5). It is estimated to affect approximately 10%–20% of the hypertensive population worldwide and is associated with an increased risk of cardiovascular disease including heart failure and stroke, kidney disease, and other complications (6–9).

The economic burden of resistant hypertension is also substantial, with increased healthcare utilization and costs associated with this condition (3). Individuals with resistant hypertension require more frequent medical appointments, diagnostic tests, and medications, resulting in higher healthcare costs. Additionally, the complications associated with resistant hypertension can result in increased hospitalizations and emergency department visits, further contributing to the economic burden of this condition.

A recent systematic review found that 14.7% of individuals with high blood pressure experience apparent treatment-resistant hypertension, while only 10.3% have been diagnosed with actual resistant hypertension (10). However, this estimate may be an underestimation as many cases of resistant hypertension go undiagnosed or are not managed adequately. Despite the availability of multiple antihypertensive medications, the management of resistant hypertension remains challenging. The reasons for this include patient-related factors such as nonadherence to medications, lifestyle factors, and comorbidities such as obesity and diabetes, as well as medication-related factors such as drug interactions, side effects, and pharmacokinetic variability (11). Moreover, the lack of consensus on the definition and management of resistant hypertension further complicates the management of this condition (12). While there is general agreement that resistant hypertension should be diagnosed after the use of three or more antihypertensive medications, there is still debate on the optimal drug regimen, the need for further investigations, and the role of invasive procedures such as renal denervation and baroreflex activation therapy.

## 2. Types of uncontrolled hypertension

### 2.1. Pseudo-resistant hypertension

Pseudo-resistant hypertension occurs when the blood pressure is elevated due to factors other than hypertension (13). This includes the white-coat effect, medication nonadherence, or improper measurement techniques. The white-coat effect is a phenomenon in which a patient's blood pressure is elevated during medical visits due to anxiety or stress (14). Medication nonadherence is a common problem that can contribute to elevated blood pressure levels. Improper measurement techniques, such as using a cuff that is too small or not allowing the patient to rest before taking the measurement, can also lead to inaccurate blood pressure readings. The diagnosis of pseudo-resistant hypertension can be confirmed through ambulatory blood pressure monitoring, which measures the blood pressure over a 24-hour period (15). Treating pseudo-resistant hypertension includes improving measurement techniques, addressing medication nonadherence, or using other medication options.

### 2.2. True resistant hypertension

True-resistant hypertension occurs when elevated blood pressure is due to a combination of factors, including genetic predisposition, lifestyle factors, medication nonadherence, and underlying medical conditions (16). True-resistant hypertension requires a comprehensive evaluation and a variety of different treatment strategies to achieve blood pressure control. Treatment approaches may include using different classes of antihypertensive medications, improving medication adherence, lifestyle modifications, and addressing underlying medical conditions. A study indicated that true resistant hypertension may be linked to inflammation and oxidative stress, and addressing these pathways could result in improved blood pressure management (17).

### 2.3. Refractory hypertension

**Figure 1:**
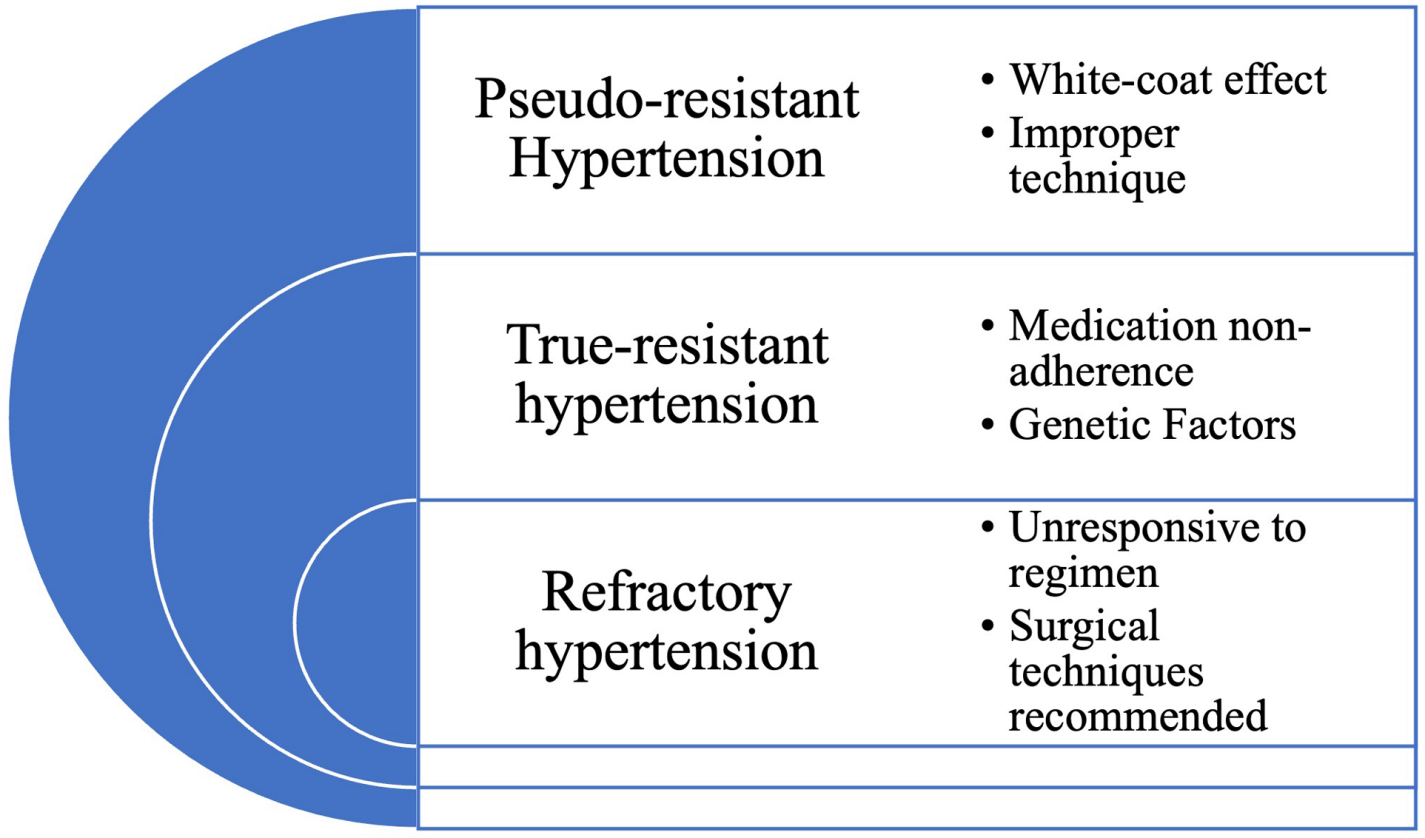
Types of uncontrolled hypertension.

Refractory hypertension occurs when the blood pressure remains elevated despite optimal treatment with multiple antihypertensive medications and lifestyle modifications (18). Refractory hypertension is a rare condition that may require more invasive interventions, such as renal denervation or baroreceptor activation therapy, to achieve blood pressure control. Renal denervation involves using radiofrequency energy to disrupt the renal nerves, which can help reduce blood pressure levels (19). Baroreceptor activation therapy involves implanting a device that stimulates the baroreceptors, which are sensors in the carotid arteries that regulate blood pressure (20). These interventions are typically reserved for patients with severe hypertension who have failed multiple treatment options. Figure 1 provides a concise summary of the different types of uncontrolled hypertension.

## 3. Etiopathogenesis

### 3.1. Nonadherence

One of the primary causes of RH is nonadherence to medications. Compliance with medication schedules is essential for hypertension management, as it has been demonstrated to enhance clinical outcomes and decrease the likelihood of cardiovascular incidents (21). It can be assessed by indirect measures like questionnaires, self-reports, pill counts, prescription refill rates, medication event monitoring systems (MEMS), and patient diaries (22, 23).

### 3.2. Complexity of medication regimens

Patients with hypertension often require multiple medications to manage their blood pressure effectively. The complexity of medication regimens can make it challenging for patients to adhere to their treatment plans. Patients may have difficulty keeping track of which medications they need to take and when, which can lead to missed doses and inconsistent blood pressure control. In addition, patients may experience confusion and frustration when they are prescribed multiple medications with different dosing schedules and instructions.

A study conducted a study to investigate the association between medication regimen complexity and medication adherence in hypertensive patients, which revealed that patients prescribed more than three antihypertensive medications had lower adherence rates compared to those prescribed fewer medications (24). Poor medication adherence can result from adverse drug events (ADEs) or insufficient knowledge and/or beliefs regarding drug therapy among patients (25).

Antihypertensive medications can cause a range of side effects, including dizziness, fatigue, and sexual dysfunction. These side effects can impact patients' quality of life and lead to medication nonadherence. Patients may also experience side effects that they perceive as intolerable or unacceptable, which can cause them to discontinue their medication without consulting their healthcare provider. Another study found that side effects were the primary reason for medication nonadherence in up to 30% of patients. The authors recommended that healthcare providers discuss potential side effects with patients when prescribing antihypertensive medications and work with patients to manage any side effects that may occur (26). The cost of medications can be a significant barrier to adherence for patients with hypertension, particularly for those who are uninsured or underinsured. Those who cannot afford their medications may also be more likely to use cost-saving strategies, such as skipping doses or reducing their medication dose, which can compromise their blood pressure control (27, 28). A study found that financial barriers to medication access are linked to lower medication adherence in patients with hypertension (29). Furthermore, inadequate social support, such as living alone or having limited social networks, can also hinder medication adherence among patients with hypertension (30, 31).

Psychological factors such as depression, anxiety, and stress can also contribute to poor medication adherence in patients with hypertension (32, 33). Healthcare providers should assess and collaborate with patients who have comorbid mental health conditions to improve medication adherence, as these patients may struggle with medication regimen management, which can impact blood pressure control (34). Patients' attitudes and beliefs towards antihypertensive medications can impact their commitment to medication therapy, with concerns about safety and efficacy or negative past experiences potentially influencing their willingness to adhere to prescribed regimens (35–37). Limited understanding or education about medications and their condition can result in medication nonadherence and poor blood pressure control among patients, who may not appreciate the significance of adhering to their prescribed medication regimen or lack comprehension about the mechanisms of their medications (38).

### 3.3. RAA system

The renin-angiotensin-aldosterone system (RAAS) regulates blood pressure and fluid balance in the body. In hypertension, the RAAS can become dysregulated, leading to increased levels of angiotensin II (Ang II) and aldosterone, which can contribute to the development of resistant hypertension (39, 40). The RAAS system is a complex cascade of hormonal and biochemical pathways that regulate blood pressure, fluid balance, and electrolyte homeostasis (40). Activation of this system occurs when the kidneys release renin in response to a decrease in sodium concentration or a drop in blood pressure (41). Angiotensinogen is cleaved by renin to produce angiotensin I (Ang I), which is subsequently converted into Ang II by angiotensin-converting enzyme (ACE) (42–44). Ang II is a potent vasoconstrictor and also stimulates the release of aldosterone from the adrenal glands (45, 46). Aldosterone promotes sodium and water retention in the kidneys, leading to increased blood volume and blood pressure (47). In hypertension, the RAAS system can become overactive, leading to increased levels of Ang II and aldosterone (44, 48, 49).

Another factor that can contribute to RAAS activation in resistant hypertension is decreased nitric oxide availability (50). The decreased NO availability and increased activation of the RAAS system in resistant hypertension contribute to the development of vascular and end-organ damage, including left ventricular hypertrophy, renal dysfunction, and increased risk of cardiovascular events such as myocardial infarction and stroke (44, 51). Reversing the effects of the RAAS system through the use of medications such as ACE inhibitors, ARBs, and MRAs has proven effective in lowering blood pressure and enhancing cardiovascular outcomes in patients with hypertension, including those with resistant hypertension (52, 53). In addition, strategies aimed at increasing NO availability, such as supplementation with L-arginine or administration of NO donors, have also been investigated as potential therapeutic options for resistant hypertension (54, 55).

### 3.4. Environmental factors

Stress is a complex environmental factor that can have both acute and chronic effects on blood pressure regulation. Acute stressors, such as mental or physical stress, can lead to transient increases in blood pressure levels, while chronic stress can lead to sustained increases in blood pressure and the development of hypertension (56). Stress leads to hypertension by causing an increase in sympathoadrenal activity, secretion of norepinephrine and epinephrine, and heightened vascular tone (57). stress can have a range of physiological effects, including an increased risk of cardiovascular disease, a reduced ability of the body's baroreceptors to regulate blood pressure, an elevated blood pressure and neuroendocrine response to acute stressors, a diminished blood pressure and neuroendocrine response to repeated stressors, and a heightened baseline blood pressure (56, 58).

Air pollution, a growing environmental concern that has been shown to be associated with an increased risk of hypertension and cardiovascular disease (59–61). Air pollution is a complex mixture of particulate matter, nitrogen oxides, and other pollutants that can have both direct and indirect effects on blood pressure regulation (59). Particulate air pollutants can increase inflammation, blood coagulability, endothelial dysfunction, acute vasoconstriction, and worsen myocardial ischemia (59).

Sleep is a crucial component of overall health, and disruptions in sleep patterns have been linked to a variety of health issues, including hypertension. Chronic sleep deprivation and poor sleep quality have been shown to be associated with increased blood pressure levels and an increased risk of developing hypertension (62). Short sleep duration, typically defined as less than 6 h of sleep per night, has been associated with an increased risk for hypertension and cardiovascular disease (63, 64).

Sleep disturbances can lead to increased sympathetic nervous system activity, which can increase blood pressure and contribute to the development of hypertension (65). Hypertension is associated with disruptions in the normal circadian rhythms of various physiological variables, such as a higher prevalence of the non-dipping pattern, a shift in the daily blood pressure profile to higher levels, irregularities in the diurnal cardiac output rhythm, and increased variability in blood pressure readings (66). Sleep deprivation induces stress, which can enhance salt appetite and reduce renal salt-fluid excretion (66).

### 3.5. Genetic factors

Genetic polymorphisms in the renin-angiotensin system have been associated with an increased risk of hypertension and resistance to antihypertensive medications (67, 68). One of the genes most commonly associated with resistant hypertension is the angiotensinogen (AGT) gene (69, 70). The AGT gene encodes a protein that is a precursor to angiotensin II, a potent vasoconstrictor that plays a critical role in the regulation of blood pressure (44). The M235T polymorphism, a polymorphism of the AGT gene, has been shown to be associated with increased plasma angiotensinogen levels and increased risk for hypertension, including resistant hypertension (71, 72). The angiotensin-converting enzyme (ACE) gene, which encodes an enzyme that converts angiotensin I to angiotensin II, has been linked to hypertension and cardiovascular disease (72). Variants in the ACE gene have been linked to increased ACE activity and elevated levels of angiotensin II, a potent vasoconstrictor that contributes to hypertension (73). The presence of the Insertion/ Deletion polymorphism in the ACE gene has been linked to a higher probability of developing essential hypertension, including resistant hypertension (74).

Studies have found that the SLC4A5 gene, responsible for encoding a sodium bicarbonate transporter in the kidney, is associated with hypertension and salt sensitivity (75). Certain genetic variations in the SLC4A5 gene were found to be correlated with the sensitivity of blood pressure to salt. Individuals with these genetic variations had reduced expression of the SLC4A5 gene and decreased renal bicarbonate reabsorption, which may contribute to the development of salt sensitivity and hypertension (68, 75).

Genetic variants in the renin gene have been associated with increased renin activity and elevated blood pressure levels (68, 76). A meta-analysis reported that a variant in the AT1R gene has also been associated with hypertension and altered response to angiotensin receptor blockers (77). The CYP11B2 gene, which encodes aldosterone synthase, has also been implicated in the development of hypertension and resistant hypertension by affecting aldosterone production and decreased response to spironolactone, a mineralocorticoid receptor antagonist commonly used in the treatment of resistant hypertension (78). Polymorphisms in this gene have been associated with increased aldosterone levels and salt sensitivity (79).

In addition to specific genetic variants, epigenetic modifications can also affect the RAAS system and contribute to the development of resistant hypertension (80). Epigenetic modifications are changes to DNA that do not alter the underlying genetic sequence but can affect gene expression. Methylation occurring in the promoter region of the ACE gene has been associated with increased ACE activity and elevated blood pressure levels (81). Similarly, DNA methylation of the aldosterone synthase gene has been linked to increased aldosterone production and hypertension (82). Genetic variants in the adrenergic receptor genes were linked to the development of resistant hypertension (83). These genetic variants may alter the responsiveness of the sympathetic nervous system to antihypertensive medications, leading to inadequate blood pressure control and the development of resistant hypertension.

### 3.6. Sympathetic nervous system

The sympathetic nervous system plays a crucial role in regulating blood pressure and cardiovascular function through its effects on the vasculature, the heart, and the kidneys. Within the brain, the renin-angiotensin system (RAS) is viewed as the primary regulator of the sympathetic nervous system (SNS) (84). The sympathetic nervous system activation can result in heightened vasoconstriction, elevated heart rate and contractility, reduced venous capacitance and increased retention of sodium and water promoting the development of hypertension (85–87). In addition to its effects on blood pressure and volume, sympathetic activation can also contribute to the development of target organ damage in patients with resistant hypertension. Sympathetic activation can promote oxidative stress and inflammation, leading to endothelial dysfunction and vascular remodelling, which are key features of hypertension (88, 89).

### 3.7. Obesity

Obesity is a common comorbidity in patients with resistant hypertension, and it is estimated that approximately 8% of individuals with resistant hypertension are obese (90). Obesity can induce modifications in the structure and function of blood vessels, promote insulin resistance and inflammation, and result in increased sodium retention, thereby posing challenges to achieving effective blood pressure control (91). Obesity is associated with increased arterial stiffness, reduced arterial compliance, and impaired endothelial function, all of which can contribute to elevated blood pressure (92, 93). Moreover, it can lead to the accumulation of fat in the walls of blood vessels, which can promote inflammation and oxidative stress and further impair vascular function (94). Adipose tissue is an active endocrine organ that produces several pro-inflammatory cytokines, including interleukin-6 (IL-6) and tumor necrosis factor-alpha (TNF-α), which can promote endothelial dysfunction and vascular remodeling (95).

Acute hyperinsulinemia can cause blood vessel dilation by releasing nitric oxide, but this process is impaired in insulin-resistant individuals with obesity or hypertension (96). Also, insulin resistance can lead to increased sympathetic nervous system activity, which can further promote hypertension and impair blood pressure control by causing sodium retention, which can contribute to hypertension through volume expansion and increased cardiac output (97).

A cross-sectional study of 5,065 patients with hypertension, found that obesity was independently associated with the presence of resistant hypertension (98). Patients with visceral obesity may be over-diagnosed with resistant hypertension due to undertreatment, underutilization of diuretics in multidrug regimens, and the “white-coat” effect being common contributing factors (98). Weight loss is a key strategy for reducing blood pressure in obese individuals and can improve vascular structure and function, reduce inflammation, and improve insulin sensitivity (99). Lifestyle interventions that promote weight loss, such as diet and exercise programs, can be effective in reducing blood pressure and improving cardiovascular outcomes in obese individuals with hypertension (100).

### 3.8. Salt intake

Most of the suggested theories for salt-induced high blood pressure involve impaired function in either the kidneys or the endocrine system, with a particular focus on the renin-angiotensin-aldosterone system (RAAS) (49, 101, 102). Another large population-based study, the National Health and Nutrition Examination Survey (NHANES), found that high salt intake was associated with an increased risk of hypertension, cardiovascular disease, and stroke (103).

Salt consumption is recognized to trigger an expansion in extracellular fluid volume, culminating in augmented blood volume and cardiac output, leading to elevated blood pressure and increased peripheral vascular resistance (49, 104, 105).

To reduce the risk of hypertension and resistant hypertension, American Heart Association recommends limiting salt intake to no more than 2,300 milligrams per day, and even lower (1,500 milligrams per day) for individuals who are at higher risk of hypertension, such as those with diabetes, chronic kidney disease, or a family history of hypertension (106).

### 3.9. Sleep apnea

Sleep apnea is a common condition characterized by recurrent episodes of partial or complete cessation of breathing during sleep. Sleep apnea is characterized by repetitive cessation of breathing during sleep, leading to intermittent hypoxia, sympathetic activation, and oxidative stress, which can contribute to the development and maintenance of hypertension (107).

Several studies have shown that sleep apnea is associated with resistant hypertension. For example, a study found that 83% of patients with resistant hypertension had moderate to severe sleep apnea (108). Another study found that 53% of patients with resistant hypertension had sleep apnea, compared to 31% of patients with controlled hypertension (109).

**Figure 2:**
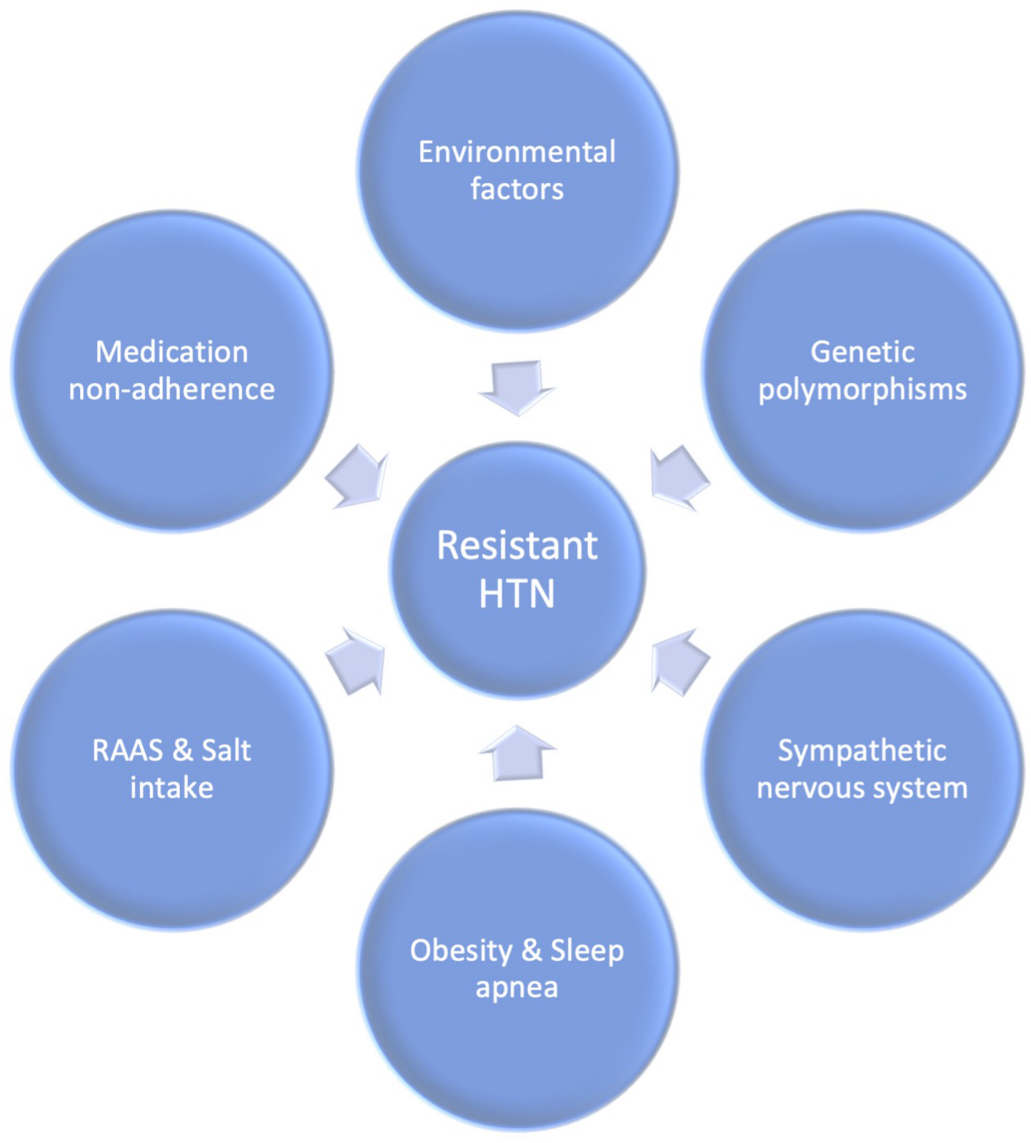
Etiology of resistant hypertension.

In obstructive sleep apnea (OSA) patients, cyclic intermittent hypoxia can trigger endothelial dysfunction and activate the renin-angiotensin and sympathetic systems (110). The release of inflammatory markers, such as hypoxia-inducible factor 1, nuclear factor kappa, endothelin-1, and various interleukins and cytokines, occurs due to the stimulation of peripheral chemoreceptors during episodes of hypoxia (107, 111, 112). These markers can damage the endothelial lining of blood vessels and increase platelet aggregation, leading to oxidative stress and further vascular endothelial damage (110). Continuous positive airway pressure (CPAP) therapy for obstructive sleep apnea has been demonstrated to decrease blood pressure in patients with resistant hypertension (110, 113, 114). Figure 2 presents a comprehensive representation of the etiological factors contributing to the development of uncontrolled hypertension.

## 4. Diagnostic challenges

The diagnosis of resistant hypertension requires careful evaluation to exclude secondary causes of hypertension and to optimize medication therapy. Recent research indicates that applying principles of chaos theory can provide new perspectives on this issue (115, 116). The approach behind this idea is based on the fact that biological systems are inherently non-linear and chaotic; therefore, minor changes in the systemic blood pressure can cause significant effects on their overall response to treatment (117, 118). This unpredictable yet significant interference adds to the challenges of diagnosing resistant hypertension, which include inadequate blood pressure control, white coat hypertension, pseudo-resistant hypertension, and medication nonadherence (119).

Clinical inertia refers to the healthcare provider's inaction to escalate treatment when the desired blood pressure target is not achieved leading to inadequate medical treatment, resulting in uncontrolled hypertension (120). Clinical inertia can be caused by several factors, including insufficient knowledge of treatment guidelines, underestimation of the patient's cardiovascular risk, and using poor justifications to avoid intensifying therapy, such as assuming the patient will not accept more medications, without discussing with the patient (120, 121).

White coat hypertension is another diagnostic challenge that clinicians may encounter when evaluating patients with resistant hypertension. White coat hypertension is defined as elevated BP in the clinic or office setting and normal BP on ambulatory or home monitoring (122–124). According to the Task Force of the Eighth International Consensus Conference on Blood Pressure Monitoring (125), ambulatory monitoring is recommended to rule out white-coat hypertension in untreated patients who meet the following criteria: (1) office blood pressure ≥140/90 mm Hg on at least three separate visits; (2) two or more blood pressure measurements taken outside of the office are <140/90 mm Hg, often utilizing home blood pressure monitoring; and (3) there is no evidence of hypertensive target organ damage. In general, the prevalence of white coat hypertension is higher in younger patients, women, and patients with mild hypertension (126).

Pseudo-resistant hypertension is a rare condition characterized by elevated BP despite the use of multiple antihypertensive medications, but it is not due to inadequate medication therapy or medication nonadherence (127). Instead, pseudo-resistant hypertension is caused by an inability to measure BP accurately, usually due to severe obesity, arm circumference, or vascular access problems (128).

The diagnosis of resistant hypertension requires a comprehensive evaluation to exclude secondary causes of hypertension and to optimize medication therapy. The American Heart Association (AHA). recommends a stepwise approach to the evaluation and treatment of patients with resistant hypertension (Table 1).

**Table 1:** A 7 step plan for evaluation and management of treatment resistant hypertension (2).

1. Confirm treatment resistance by checking that the patient's office blood pressure is ≥140/90 mmHg despite taking ≥3 blood pressure medications at optimal doses, preferably including a diuretic.
2. To exclude pseudoresistance, check if the patient is adherent to an optimal treatment regimen and whether their out-of-office blood pressure is elevated.
3. Identify and address lifestyle factors that contribute to hypertension, such as obesity, physical inactivity, excess alcohol consumption, and high salt intake.
4. Discontinue or reduce interfering substances, including NSAIDs, sympathomimetics, oral contraceptives, erythropoietin, and non-prescription weight loss supplements containing ephedra.
5. Screen for secondary hypertension causes, including obstructive sleep apnea, primary aldosteronism, chronic kidney disease, renal artery stenosis, and pheochromocytoma.
6. To optimize pharmacologic treatment, consider enhancing diuretic therapy and using alpha1 and beta1 adrenoceptor blockade in patients with an otherwise optimal regimen, while avoiding combining beta-blockade with non-dihydropyridine CCB.
7. If the patient's blood pressure remains uncontrolled after six months or if there are known or suspected secondary causes of hypertension, it is recommended to refer them to a hypertension specialist for definitive evaluation and treatment.

The AHA recommends the use of ambulatory monitoring or home BP monitoring to confirm the diagnosis of resistant hypertension and to monitor BP control (2). Ambulatory monitoring involves the use of a portable BP monitoring device that is worn for 24 h, and it provides multiple BP measurements throughout the day and night (2). Home BP monitoring involves the use of a BP monitoring device that is used by the patient at home, and it provides multiple BP measurements over several days (129).

The appropriate diagnostic tests, such as polysomnography, aldosterone-renin ratio, or renal artery imaging, should be used to confirm the diagnosis and to guide treatment (130).

## 5. Treatment options

### 5.1. Lifestyle modifications

Resistant hypertension is a challenging condition to manage, and it often requires a combination of pharmacological and lifestyle interventions. While pharmacological treatments are effective in controlling blood pressure in most patients, some patients may not achieve adequate blood pressure control despite multiple medications. Lifestyle changes, such as adopting a healthy diet, increasing physical activity, and reducing stress, can play a critical role in managing resistant hypertension (131). The AHA recommends lifestyle modifications, such as a low-salt diet, weight loss, regular exercise, and moderation of alcohol consumption, for all patients with hypertension (2, 130).

According to the TRIUMPH trial, lifestyle modifications, including weight loss, physical activity, and dietary changes, were associated with a significant reduction in blood pressure among patients with resistant hypertension (132). The study also found that these changes led to improvements in overall cardiovascular health and a reduced risk of adverse cardiovascular events.

There is a growing body of evidence supporting the role of lifestyle changes in managing resistant hypertension. For example, a meta-analysis found that lifestyle interventions, including weight loss, reduced sodium intake, and increased physical activity, were effective in reducing blood pressure levels in patients with resistant hypertension. The study also highlighted the importance of individualized lifestyle interventions tailored to each patient's unique needs and circumstances (133).

Regular physical activity can improve cardiovascular health by reducing inflammation and improving lipid profiles (121). A healthy diet, rich in fruits, vegetables, and whole grains, can also reduce the risk of other chronic diseases, such as diabetes and cancer (134). The Dietary Approaches to Stop Hypertension (DASH) is a dietary intervention that has been shown to be effective in reducing blood pressure in patients with resistant hypertension in several trials (100, 135–137). The DASH diet emphasizes the consumption of fruits, vegetables, whole grains, and low-fat dairy products while limiting the intake of processed foods, saturated fats, and added sugars (138). A systematic review and meta-analysis of randomized controlled trials found that the DASH diet was associated with a significant reduction in blood pressure (139). Similarly, a Mediterranean-style diet, which emphasizes the consumption of fruits, vegetables, whole grains, fish, and healthy fats, has been found to be effective in reducing blood pressure in patients with resistant hypertension. A randomized controlled trial found that participants following a Mediterranean-style diet experienced significant reductions in blood pressure compared to those following a low-fat diet (140).

Regular physical activity has been found to have a beneficial effect on blood pressure control and can be an effective non-pharmacological intervention in managing resistant hypertension. Several studies have shown that exercise, both aerobic and resistance training, can lead to reductions in both systolic and diastolic blood pressure in patients with resistant hypertension (121, 134, 141).

One study found that a combination of aerobic and resistance exercise performed three times per week for 12 weeks resulted in significant reductions in systolic and diastolic blood pressure by possibly changing the vascular responses in patients with resistant hypertension (141).

### 5.2. Pharmacological intervention

Pharmacological interventions for resistant hypertension have been the subject of intense research, with several classes of drugs showing promise in lowering blood pressure in these patients. One class of drugs that has garnered considerable attention in recent years is mineralocorticoid receptor antagonists (MRAs). MRAs, such as spironolactone and eplerenone, have been shown to reduce blood pressure in patients with resistant hypertension, with spironolactone being the most extensively studied in this population. MRAs have been found to reduce mortality in patients with heart failure with reduced ejection fraction (HFrEF) (142) and are recommended for patients with resistant hypertension (8) and secondary hyperaldosteronism (143).

A sequential monotherapy approach that targets multiple physiological pathways is utilized for optimizing blood pressure control by identifying the most effective antihypertensive agents for individual patients (144). This approach takes into account the variability in individual patient responses to different medications and aims to optimize treatment outcomes by tailoring therapy to the specific needs of each patient (144). Pharmacological interventions for resistant hypertension have undergone extensive research, with promising results observed for various drug classes in lowering blood pressure in these patients. Notably, the use of drug combinations as a therapeutic strategy has gained significant attention. The use of two or more drugs with complementary mechanisms of action can potentially enhance their efficacy by addressing several causative factors while minimizing adverse effects (145). The effectiveness and safety of combination therapy depend on several factors such as patient characteristics, drug interactions, and adherence. In the past, concerns existed regarding the association of different drug classes leading to a profound reduction in blood pressure levels. The combination of blood pressure-lowering drugs from diverse classes has been shown to be approximately five times more effective compared to doubling the dose of a single drug (146–148). A meta-analysis findings indicate that doubling the dose of a single blood pressure-lowering drug resulted in a modest additional decrease of 4% in coronary heart disease events and 5% in stroke incidence. However, combining two drugs from different classes led to a more substantial reduction in blood pressure of approximately 9 mm Hg, corresponding to an additional reduction of 15% in coronary heart disease events and 19% in stroke incidence (146). The antihypertensive effectiveness of renin-angiotensin-aldosterone system (RAS) blockers is enhanced by the thiazide-induced increase in sodium excretion, as it stimulates renin release (148). Concurrently, RAS blockers attenuate the potassium depletion induced by thiazide diuretics, thereby minimizing the likelihood of hypokalemia (149). An open-label study found that a combination drug of an angiotension receptor blocker (ARB) olmesartan and a calcium channel blocker (CCB) azelnidipine was effective in reducing both systolic and diastolic blood pressure in patients with resistant hypertension in both office and at home (150). The study also noted that the combination therapy was generally well-tolerated and did not result in any significant adverse events. A comprehensive meta-analysis encompassing 27 studies and nearly 50,000 hypertensive patients with type 2 diabetes mellitus revealed that the utilization of both a renin-angiotensin system inhibitor (RASI) and a calcium antagonist exhibited remarkable efficacy in reducing mortality rates when compared to alternative treatment approaches such as monotherapy or different combinations (151). These also have been demonstrated to have advantageous effects beyond their blood pressure-lowering impact, such as metabolic, anti-inflammatory, reno-protective, and hemodynamic improvement, while combinations including diuretics have yielded superior outcomes in individuals with heart failure (152). Direct renin inhibitor Aliskiren can be used as a fourth or fifth line drug in resistant hypertension, significantly reducing both systolic and diastolic blood pressure (153). A study conducted on patients with true resistant hypertension revealed that despite receiving a combination treatment involving four drugs, a significant proportion of individuals had uncontrolled blood pressure, highlighting the need for more aggressive interventions and exploration of non-pharmacological approaches such as renal denervation, electric and magnetic stimulation (152, 154).

### 5.3. Surgical interventions

Although non-pharmacological and pharmacological interventions are the mainstay of treatment, some patients may not achieve adequate blood pressure control despite these interventions. In such cases, surgical intervention may be considered. Renal denervation (RDN) is a minimally invasive surgical intervention that has emerged as a potential treatment option for resistant hypertension. The procedure involves the use of radiofrequency energy to disrupt the renal sympathetic nerve fibers, leading to a reduction in sympathetic nervous system activity and subsequent blood pressure reduction (155). Several clinical trials have evaluated the safety and efficacy of RDN in patients with resistant hypertension. The Symplicity HTN-1 trial was the first to demonstrate the feasibility of RDN and showed a significant reduction in blood pressure at 6 months post-procedure (156). The subsequent Symplicity HTN-2 trial, which was a randomized controlled trial, also showed a significant reduction in blood pressure in patients treated with RDN compared to those treated with usual care (157). However, a subsequent randomized controlled trial, the Symplicity HTN-3 trial, failed to demonstrate a significant reduction in blood pressure in patients treated with RDN compared to those treated with a sham procedure (19). This led to a temporary suspension of RDN procedures in some countries and raised questions about the efficacy of the procedure.

Despite these conflicting results, a recent meta-analysis has suggested that RDN may be effective in reducing blood pressure in patients with resistant hypertension. The meta-analysis of 11 controlled studies on renal denervation (RDN) in resistant hypertension found that RDN significantly reduced systolic and diastolic blood pressure respectively (158). The analysis also showed that the beneficial effect of RDN on blood pressure was sustained over a long-term follow-up period of up to 36 months. However, there have been reports of renal artery stenosis, renal artery dissection, and renal infarction following RDN, emphasizing the importance of careful patient selection and monitoring (159). Patient compliance with RDN appears to be high, with a recent study reporting a 96% 12-month retention rate in patients treated with RDN (160). However, long-term follow-up is needed to determine the durability of the blood pressure-lowering effect of RDN and its impact on cardiovascular outcomes.

Transcranial direct current stimulation (tDCS) (161) is an emerging minimally invasive technique that has the potential to reduce blood pressure by modulating the vagal action (162, 163). It involves applying a direct current of low intensity to the scalp, which can reach the cortical regions of the brain by penetrating the skull (164–166). tDCS has demonstrated promising potential as a treatment modality for neurological disorders such as depression or Parkinson's disease (167). Previous research has demonstrated an elevation in sympathetic discharge (168) and enhanced heart rate variability (169, 170) following tDCS. Therefore, there may be broader benefits associated with using tCDS extending to treating refractory hypertension. The initial investigation on the effects of tDCS on systemic blood pressure revealed a significant reduction as measured by 24 h ABPM, potentially mediated by cardiovascular autonomic balance (171). While it has shown promise in treating various neurological conditions like depression and chronic pain, its efficacy in addressing hypertension requires further study.

Transcranial magnetic stimulation (TMS) utilizes electromagnetic induction to produce a magnetic field capable of traversing the skull and accessing the brain (162). Consequently, this magnetic field induces an electric field in the superficial cortical regions, resulting in the excitation of neurons and axons (172). Animal studies have demonstrated a reduction in blood pressure using repetitive TMS (rTMS)… Although preliminary studies demonstrate promising results, larger-scale trials are essential to fully evaluate its efficacy and potential side effects. Additionally, researchers must address questions about optimal dosing protocols and electromagnetic coil placement before recommending widespread use.

## 6. Complications

Resistant hypertension is a serious condition that can lead to various complications, including organ damage. The failure to control blood pressure can lead to various complications and hazards, including increased risk of cardiovascular disease, stroke, kidney damage, and other related health issues (173, 174).

### 6.1. Increased risk of cardiovascular disease

Resistant hypertension is associated with an increased risk of cardiovascular disease, including stroke, heart attack, and heart failure. Patients with resistant hypertension have a significantly higher risk of stroke compared to patients with controlled hypertension which may be due to the increased risk of stroke may be due to the greater severity of hypertension and the presence of other cardiovascular risk factors in patients with resistant hypertension (173, 175).

### 6.2. Chronic kidney disease

CKD, which is characterized by an estimated glomerular filtration rate (GFR) below 60 ml/min/1.73 m^2^ and proteinuria, are significant predictors of resistant hypertension (RHT) (2, 9, 173). The uncontrolled high blood pressure can damage the kidneys over time by causing injury to the blood vessels in the kidneys, leading to a reduction in their ability to filter waste and excess fluids from the body. Approximately 38% of patients with resistant hypertension also had CKD, which was associated with an increased risk of cardiovascular events and mortality (176).

### 6.3. Adverse drug effects

Adverse drug events (ADEs) may occur in patients with resistant hypertension who are on multiple antihypertensive medications, ranging from mild symptoms such as dizziness and fatigue to more severe complications such as hypotension, electrolyte imbalances like hyperkalemia, renal dysfunction, acute kidney injury (AKI), and orthostatic or diastolic hypotension (177). The risk of ADEs may also be increased in older adults and those with comorbidities such as chronic kidney disease or diabetes. The use of angiotensin-converting enzyme (ACE)-inhibitors/angiotensin II receptor blockers (ARBs), potassium-sparing diuretics, and β-blockers has been associated with hyperkalemia, while ACE-inhibitor/ARB therapy has been most commonly reported to cause acute kidney injury (AKI), and orthostatic hypotension has been evaluated in patients taking ACE-inhibitor/ARB, β-blocker, or calcium-channel blocker therapy (177).

### 6.4. Poor quality of life

Resistant hypertension can have a significant impact on a patient's quality of life. The need for multiple medications and frequent adjustments can lead to medication side effects, increased healthcare costs, and reduced ability to engage in physical activity. Patients may also experience increased anxiety and depression related to the difficulty of managing their condition. Additionally, the burden of monitoring and managing their blood pressure can lead to decreased social interactions and a reduced sense of control over their health. A study found that patients with hypertension had lower health-related quality of life (HrQoL) compared to those without hypertension (178). Reductions were observed across all domains of the survey, including physical functioning, role physical, bodily pain, general health, vitality, social functioning, role emotional, and mental health, as well as in both the physical and mental component summary scores.

## 7. Conclusion and future directions

This review has examined the current state of knowledge regarding resistant hypertension, with a focus on the epidemiology, pathophysiology, diagnosis, and management of this condition. We have discussed the various definitions of resistant hypertension and the prevalence and risk factors associated with this condition. We have also explored the underlying mechanisms involved in resistant hypertension, including alterations in the renin-angiotensin-aldosterone system, sympathetic nervous system activation, and endothelial dysfunction. Diagnosis of resistant hypertension involves careful evaluation of patients’ medication regimens, as well as identification and management of contributing factors such as obesity, sleep apnea, and secondary causes of hypertension. We have also reviewed the various pharmacological and non-pharmacological interventions that can be used to manage resistant hypertension, including lifestyle modifications, medication adjustments, renal denervation, and other emerging therapies.

Resistant hypertension remains a significant clinical challenge, and research efforts should continue to explore new approaches to its management. One potential avenue for future research is the use of novel pharmacologic agents or combinations of existing drugs to improve blood pressure control. For example, new classes of drugs targeting the renin-angiotensin-aldosterone system or the sympathetic nervous system are currently in development and may prove effective in treating resistant hypertension.

Another area of potential investigation is the use of non-invasive neuromodulation techniques, such as transcranial magnetic stimulation (TMS) or transcranial direct current stimulation (tDCS). These approaches have shown promise in animal studies as well as small sample human studies and may provide a non-pharmacologic alternative with fewer complications like infections and reduced recovery time, for patients with resistant hypertension. When traditional treatments fail to achieve desired results, non-invasive techniques such as transcranial direct current stimulation (tDCS) and transcranial magnetic stimulation (TMS) provide an alternative course of action by selectively modulating the autonomic nervous system to regulate and reduce blood pressure. By taking advantage of TMS' ability to inhibit sympathetic outflow from distinct parts of the brain—resulting in minimal systemic side effects commonly seen with conventional anti-hypertension medications—it might be possible to lower patients' blood pressures more effectively. These minimally invasive techniques offers a more cost-effective and safer option compared to other invasive interventions available today since it does not require any medication or surgery. Incorporating these techniques into clinical practice necessitates the development of consistent protocols, the selection of suitable patients, and the identification of biomarkers or neuroimaging techniques to anticipate treatment response. Precision medicine approaches could aid in customizing minimally invasive interventions for individual patients, resulting in improved outcomes. The use of innovative approaches, such as machine learning and artificial intelligence, may also offer new opportunities for improving the diagnosis and management of resistant hypertension.

Finally, advances in precision medicine may also offer new opportunities for personalized management of resistant hypertension. By using genetic and other biomarkers to identify patients who are most likely to respond to specific treatments, clinicians may be able to tailor therapy and achieve better outcomes. Another potential application of precision medicine in resistant hypertension is the use of digital health technologies to monitor blood pressure and other physiological parameters in real time. Wearable devices and mobile applications could be used to collect data on blood pressure, heart rate, physical activity, and other relevant factors, which could then be analyzed to identify patterns and predict the risk of complications (Table 2). This information could be used to adjust treatment regimens and optimize the management of resistant hypertension.

**Table 2:** Future directions in resistant hypertension.

Development of novel pharmacologic agents or drug combinations targeting the renin-angiotensin-aldosterone system and sympathetic nervous system
Investigation of non-invasive neuromodulation techniques, such as transcranial magnetic stimulation and transcutaneous vagal nerve stimulation
Utilization of machine learning and artificial intelligence to improve diagnosis and management of resistant hypertension
Advancement of precision medicine for personalized management of resistant hypertension, including the use of genetic and biomarker-based approaches
Implementation of digital health technologies, such as wearable devices and mobile applications, for real-time monitoring and optimization of treatment regimens

Meanwhile, healthcare providers must remain vigilant in identifying patients with resistant hypertension and implementing appropriate interventions to reduce cardiovascular risk. Further research is needed to advance our understanding of this condition and to identify new and innovative approaches for its management. Through continued research and collaboration, we can work towards improved outcomes and better health for patients with resistant hypertension.
